# Nasal Septal Deviation After Surgically Assisted Rapid Maxillary Expansion

**DOI:** 10.1007/s12663-021-01529-w

**Published:** 2021-02-26

**Authors:** Francesco Seidita, Pedro Henrique de Azambuja Carvalho, José Cleveilton Dos Sántos, Giovanni Dell’Aversana Orabona, Luigi Califano, Mário Francisco Real Gabrielli, Valfrido Antonio Pereira Filho

**Affiliations:** 1 Department of Oral and Maxillofacial Surgery, University Hospital Federico II, Via Sergio Pansini, 5, Naples, Italy; 2 Department of Oral and Maxillofacial Surgery, Dental School of Araraquara, São Paulo State University (UNESP), São Paulo, Brazil

## Abstract

**Background and Aim:**

Surgically assisted rapid maxillary expansion (SARME) is a surgical technique widely used to correct deficiency of the transverse maxillary dimension. Although some studies investigated the effect of SARME on nasal and facial alterations, there is no evidence that correlates nasal septal deviation (NSD) to SARME as a possible postoperative sequel. The aim of this study is to address and quantify possible variations in the position of the nasal bony septum after SARME and identify any NSD as a postoperative outcome of this surgical technique.

**Patients and Methods:**

This is a retrospective study, conducted at the Department of Oral and Maxillofacial Surgery of the University Hospital of Araraquara (Unesp, faculty of dentistry), SP, Brazil. Twenty-nine patients who underwent SARME were studied; every patient was evaluated by cone-beam computerized tomography (CBCT) before (*T0*) and six months after surgery (*T1*), and we collected the variation of nasal septal position by measuring the distance between the bony septum and the nasal lateral wall. Our measurements were carried out at the level of the head, midpoint and tail of the inferior turbinate.

**Results:**

A mean NSD ranging from 0.4 to 1.2 mm was measured, and it is more pronounced at the anterior part of the bony septum. Twenty-seven patients (93.1%) presented minor changes in bony septum position; in 2 cases (6.8%), a significant NSD was found (*p* < 0.05).

**Conclusion:**

A variation of bony nasal septum position can be expected in any direction after SARME, and it is more pronounced at anterior portion.

## Introduction

Nasal septal deviation (NSD) is defined as the deviation of the bony or cartilaginous septum or both from the facial midline. This condition may cause significant nasal obstruction, affecting nasal airflow and increasing nasal airway resistance [1, 2]. It is reported that the prevalence of NSD is 1–20% in newborns [3, 4] and 20% at school age [5]. In adults, it ranges from 65 to 80% [6, 7].

Surgically assisted rapid maxillary expansion (SARME) is a surgical technique widely used to correct transverse discrepancies of the jaws greater than 5 mm, in patients presenting transverse maxillary deficiency, posterior cross-bite, anterior teeth crowding and wide buccal corridor [8]. The deficiency of the transverse maxillary dimension is caused by early completion of the palatal suture fusion, and it must be surgically treated if previous orthodontic therapy has been ineffective or not possible [8]. Most commonly reported complications and sequelae of SARME are postoperative pain, paresthesia, swelling, asymmetric expansion and tooth-related problems, such as loss of vitality, root resorption, mobility and bone loss between incisors [8].

Some trials investigated the effect of orthodontic rapid maxillary expansion on nasal septal changes and reported significant deviations of the nasal septum after orthodontic treatment [9, 10]; others reported few and nonsignificant alterations of nasal septum [11, 12] or did not find any NSD [13]. Although some studies investigated the effect of SARME on nasal and facial alterations [14–18], there is no evidence that correlates NSD to SARME as a possible postoperative outcome.

The aim of this study is to address and quantify possible variations in the position of the bony nasal septum after SARME and identify any deviation of the septum as a postoperative outcome of this surgical technique.

## Materials and Methods

This is a retrospective study, conducted according to the rules of the Declaration of Helsinki and approved by the Local Research Ethics Committee.

### Sample Selection

Medical records of 29 patients with maxillary transverse deficiency greater than 7 mm, operated in the last 6 years, were included in the study. Syndromic patients, those who had additional maxillary surgery and patients without complete tomographic records, were excluded.

All patients received the SARME procedure, by two surgeons (V.A.P.-F. and E.S.G.), using the same surgical technique: maxillary Le Fort I bilateral osteotomies, disjunction of the anterior nasal spine from the nasal septum, pterygomaxillary disjunction and midpalatal raphe osteotomy. The expansion screw activation protocol was 0.75 mm per day, divided into three installments of 0.25 mm and initiated after 7 days postoperatively, until cross-bite correction. After expansion, appliances were blocked and left in place for 4 months as contention. After that, appliances were replaced by a transpalatal arch.

### CBCT Acquirement and Orientation

Every patient was evaluated by cone-beam computerized tomography (CBCT) at two different time points: preoperative (T0) and six months postoperative (T1). All CBCTs were obtained in a i-CAT scanner (Imaging Sciences International, Hatfield, PA, USA), adjusted to 120 kVp, 36 mA, 0.3 mm voxels and a FOV of 17 cm × 23 cm. All tomography were standardized by repositioning the 3D volume reconstruction using the software Dolphin Imaging 11 (Dolphin Imaging and Management Solutions, Chatsworth, CA), with the Frankfurt plane parallel to the ground, a line passing through both inferior orbital rims parallel to the ground and rotation correction by placing the zygomatic arch insertion in the same position bilaterally.

### Septal Position Measurement

To evaluate changes in the position of the nasal septum, the distance between the septum and the lateral walls of the nasal fossa, at the level of the insertion of the bony skeleton of the inferior turbinate, was projected on the horizontal plane and measured. Considering that most of reference points below the inferior turbinate could present short levels of remodeling, we developed a method performing all measurements immediately above inferior turbinate, avoiding the osteotomy line and the basal part of nasal fossa.

**Fig. 1:**
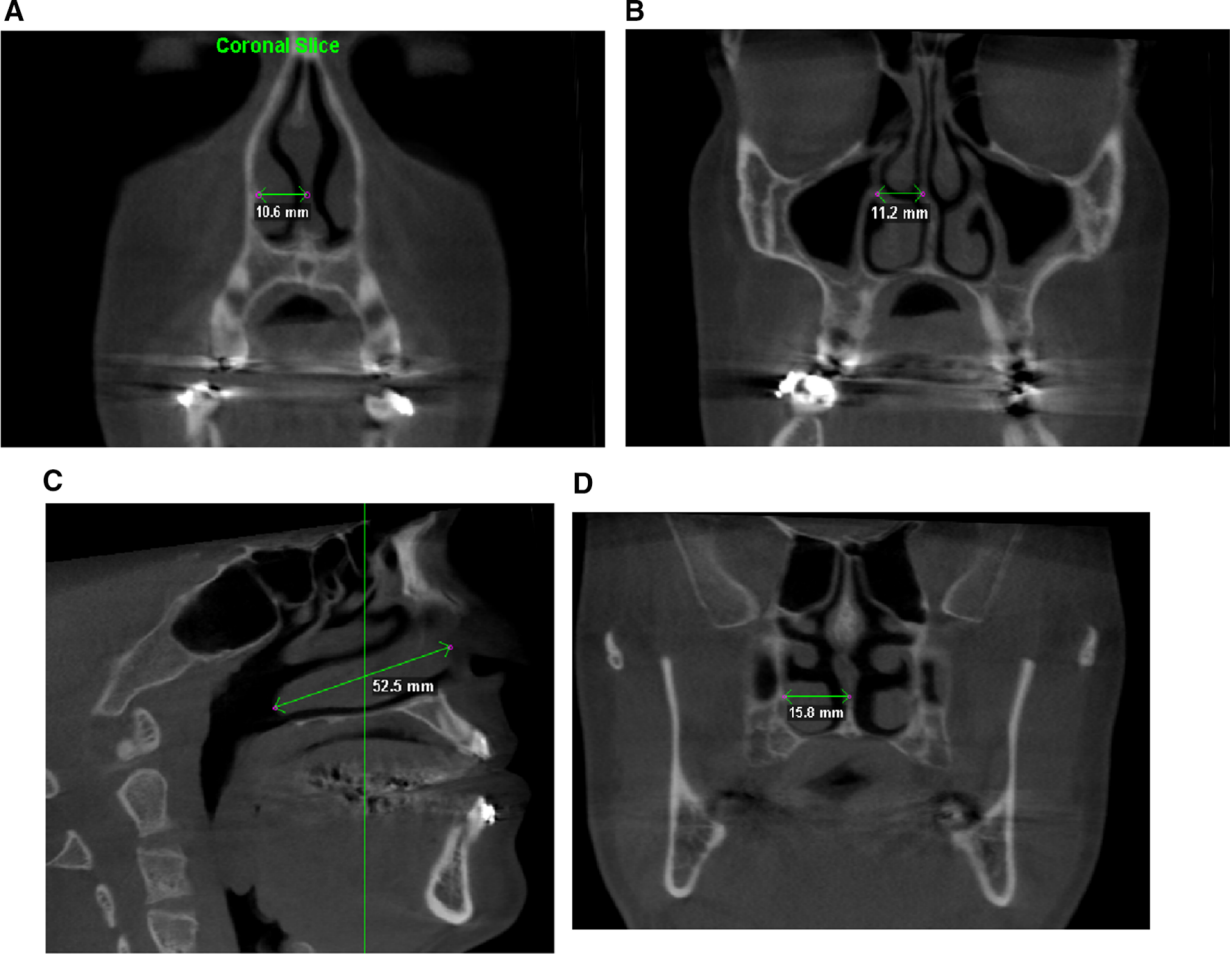
Illustration of the measurement method on the CT

For a complete study of the nasal septum position on the anteroposterior plane, measurements were carried out at the level of the head of the inferior turbinate (Fig. 1a), at the level of its midpoint, previously identified in the sagittal plane (Fig. 1b, c) and at the level of the tail of the inferior turbinate (Fig. 1d).

The distance between the most central point of the septum in the coronal section and the lateral wall of the nose, exactly at the point of insertion of the skeleton of the inferior turbinate, was obtained.

To ensure that the method is valid and repeatable, all measurements were performed by two different operators in two different timepoints, with and Interclass Correlation Index of 0.9 (high correlation), and intraclass correlation > 0.95.The average of both examiners was used as final measure for each reference point.

### Septal Deviation Estimate

The difference between T0 and T1 measurements for each side was calculated for each patient. In addition, the absolute values for all the distances between the septum and both right and left nasal walls were measured and tabulated. The difference between all T0 and T1 absolute values was obtained.

Since the nasal septum is not always centered in relation to the right and left nasal walls and considering that the position of the nasal walls may be altered by palatal expansion, the difference between the right and left distances in T0 and T1 was calculated. The difference (*Delta*) between those two parameters was chosen as the most accurate index of alteration of the nasal septum position after expansion, in relation to the lateral nasal walls.

To better understand how to get the Delta value, the following mathematical expression can be used:

$${\text{Delta}} = \left[ {\left( {T0{\text{ right distance}}} \right) - \left( {T1{\text{ right distance}}} \right)} \right] - \left[ {\left( {T0{\text{ left distance}}} \right) - \left( {T1{\text{ left distance}}} \right)} \right]$$

Mathematically, the same value can also be obtained by reversing the order of the sub-drawers, but with the opposite sign:

$${\text{Delta}} = \left[ {\left( {T0{\text{ right distance}}} \right) - \left( {T0\,{\text{left distance}}} \right)} \right] - \left[ {\left( {T1{\text{ right distance}}} \right) - \left( {T1{\text{ left distance}}} \right)} \right]$$

The positive or negative sign is not relevant, as it is indicative only of the deviation side and not of the extent of the deviation.

The data were tabulated into the statistical software SPSS (IBM SPSS Statistics for Windows, version 20.0. IBM Corp., Armonk, NY, USA). Normality tests were applied to the measurements of right and left pre- and postoperative septal position and to the delta of septal positional change. All the data presented normal distribution (Shapiro–Wilk: 0.950–0.980, *p* > 0.05). Paired t tests were applied to compare pre- and postoperative septal position, and standardized T-distribution was plotted to analyze the delta of septal positional changes.

## Results

The mean maxillary expansion was 8.1 ± 1.2 mm. At the level of the head of the inferior turbinate, on the right side, there was a mean preoperative distance of 11.25 mm to the lateral nasal wall. The mean postoperative distance was 11.48 mm, an average difference of 0.23 mm. On the left side, the mean pre- and postoperative distances were, respectively, 10.49 mm and 10.47 mm, without statistically significant differences.

At the level of the middle portion of the inferior turbinate, on the right side, there was a mean distance of 12.6 mm before surgery and 12.9 mm after surgery, with a difference of 0.34 mm. On the left side, pre- and postoperative distances were, respectively, 11.2 mm and 11.37 mm, with a mean difference of 0.15 mm).

At the level of the turbinate tail, on the right side, there was a mean distance of 13.44 mm before surgery and 13.21 mm after surgery, with a mean difference of 0.22 mm. On the left side, mean distances were, respectively, 13.04 mm and 13.54 mm, with an average difference of 0.5 mm.

**Fig. 2:**
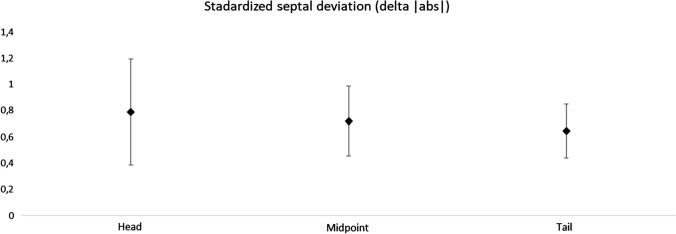
Standard septal deviation in the three examined positions, presented in mean and 95% confidence interval for absolute deviation

The mean of all the lateral nasal wall—nasal septum distances, including the right and left side together, is summarized in Table 1 (see also Fig. 2). Neither at the head, midpoint or tail of the turbinate, there were statistically significant differences between pre- and postoperative values.

**Table 1:** Septal position, in millimeters, in relation to nasal lateral walls at the level of inferior turbinate

	Pre-OP	Post-OP	*P* value
Head	0.755 (1.837)	1.021 (2.695)	0.302
Mid	1.4 (3.42)	1.569 (3.294)	0.465
Tail	0.397 (1.125)	− 0.317 (1.107)	0.001
Head|abs|	1.472 (1.311)	2.041 (2.007)	0.02
Mid|abs|	2.869 (2.281)	2.928 (2.127)	0.763
Tail|abs|	1.017 (0.597)	0.938 (0.647)	0.624
*Delta*			
Head	− 0.266 (1.36)		< 0.05
Mid	− 0.169 (1.227)		< 0.05
Tail	0.714 (1.013)		< 0.05
Head|abs|	0.79 (1.106)		< 0.05
Mid|abs|	0.721 (0.733)		< 0.05
Tail|abs|	0.645 (0.564)		< 0.05

Analysis of pre- and postoperative position and changes in septal position (Delta). [*Mean (Standard Deviation)*]

^*^At a significance level of 95%

^**^Signal indicates the deviation settled to right or left, in relation to midline

Table 1 also shows the Delta index between pre- and postoperative periods. It was on average 0.79 mm at the head of the inferior turbinate (*P* < 0.05), 0.72 mm at the midpoint (*P* < 0.05), and 0.64 mm at the tail (*P* < 0.05) (see also Fig. 2). ICC was highly significant at 99.6%.

## Discussion

Transverse maxillary deficiency is a skeletal deformity characterized by a constricted maxilla, showing unilateral or bilateral cross-bite and crowded teeth [19]. SARME is the recognized treatment approach for that situation. Its primary goal is to achieve skeletal expansion, rather than dental expansion, and to minimize dental tipping by separating the midpalatal and lateral maxillary sutures [20]. This surgical technique does not only determine a palatal expansion, but also causes craniofacial structural changes, such as the increase in nasal cavity width and nasal volume [21–24].

In fact, even if SARME is an effective and relatively safe technique [25, 26], many soft and hard tissue changes, such as nasal soft tissue changes, may occur after the surgical procedure and distraction phase [27]. It is important to analyze the nasal region in particular, being one of the most important aesthetic parts of the face [28, 29]. Several studies have reported that SARME affects the nasal area, in particular causing alar base widening, which is aesthetically undesirable in patients with a wide nose [14–18]. The increase in nasal volume after expansion generally determines a functional improvement in respiration. Patients with maxillary transverse deficiency, who underwent SARME, experienced a subjective improvement or did not have worsening of nasal obstruction after follow-up for 6 months [30]. However, to our knowledge, there are no evidences in the literature that correlate NSD to SARME as a possible postoperative outcome.

For measurements, after standardization and orientation of the tomography, we chose the level of the inferior turbinate, because this is above the Le Fort 1 osteotomy and at the same time it is a relatively caudal portion of the nasal fossa, where there would be lower resistance to the deviating forces, as compared to the higher, more cranial portions of the septum, which are anchored to the skull base. Furthermore, the head of the inferior turbinate, its tail and midpoint are easily identifiable landmarks, so the method is reproducible, independently of the operator.

There were statistically significant variations of the bony septum position. Even if there was not statistical difference for the absolute wall-septum distance values, for all the three studied landmarks the Delta index was significant (Table 1). In particular, in two patients out of 29, we noticed an important deviation of the nasal septum. The first is a 30-year-old woman with a left wall-septum distance of 8.8 mm at the inferior turbinate head, which became 5.2 mm after expansion. The second is a 40-year-old woman with a right wall-septum distance at the inferior turbinate midpoint of 11.1 mm which became 12.6 mm, while the left side went from 9.9 to 8.2 mm. In both cases, the absolute difference in the wall-septum distance after expansion does not show a large variation, but the *Delta* index was statistically different (5.5 mm in the first case at the turbinate head point and 3.2 mm in the second case at the turbinate midpoint).

Another patient presented the opposite situation. The wall-septum distance increased by almost 3 mm both to the right and to the left sides. It does not mean that the septum has changed, but that the nasal cavity has undergone an expansion process. In fact, despite the big variation in wall-septum distance, the *Delta* index in this case was not significant.

The examples of those three patients demonstrate that the direct differences in the wall-septum distances, before and after maxillary expansion, are not indicative of NSD, because the nasal walls may undergo alterations after SARME. The *Delta* between the pre- and postoperative measurements on the one side, compared to the obtained for the other side, is efficient to represent the changes in the bony septal position. The *Delta* value can be negative or positive, depending on how the septum has moved. All the numbers were considered with a positive sign, to calculate the mean amount of NSD, regardless of the side of the septal deviation.

**Fig. 3:**
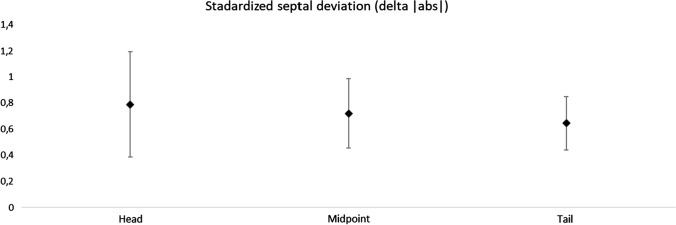
Standardized septal deviation and confidence interval of 95% (IC95)

The average NSD value was 0.79 mm at the point of the lower turbinate head, 0.72 mm at the turbinate midpoint and 0.65 mm at the tail. It is interesting to note that the deviation is greater anteriorly. In most patients, small changes in the bony septum position were not symptomatic. Despite the absence of a clinically detected NSD, small oscillations of the position of bone septum have been found in this sample, without difference between sides. Considering a confidence interval of 95% (IC95), it is possible to assume that SARME, in this study sample, produced an NSD between 0.4 and 1.2 mm, more pronounced at the anterior part of bony septum (Fig. 3).

In this sample, there was an increase in the mean transverse dimension of the nasal fossa at the level of the inferior turbinate, where measurements were taken, after SARME. In fact, despite this level is above the Le Fort 1 osteotomy, the expansion of the bone segments below the osteotomy line creates a remodeling of the nasal walls that extends higher. The influence of SARME on the nasal cavity is due to the separation of the nasal lateral walls, although only at their inferior portion. It is interesting to notice that the remodeling also affects the bone above the osteotomy line. The increase in the distance between the nasal lateral walls enlarges the cross-sectional area, increasing the nasal volume and facilitating breathing [20]. Warren et al. [31] reported that the nasal volume increased 55% after SARME.

There were no side differences in the deviation of the nasal septum. In fact, at the head of inferior turbinate there were 15 deviations to the left and 14 to the right, at the midpoint 16 to the left and 13 to the right, and at the tail of inferior turbinate 8 to the left and 21 to the right. It is probably a coincidence that most of the posterior deviations were to the right side. In most cases, rather than a real deviation to a side, small subclinical oscillations of the position of the bone septum occurred.

In summary, 27 patients (93.1%) presented minor changes in bony septum position, which were not symptomatic. Despite the absence of a clinically detected NSD, small oscillations in the position of bony septum were found throughout the sample, without side differences. In 2 cases (6.8%), a significant NSD was found. The deviation was clinically manifested and perceived by the patients, who noticed reduction of the air flow at the affected side.

Our method proved to be effective for the study of the positional changes of the bony nasal septum. Deviations of the cartilaginous septum were neither measured nor clinically identified: this is a limitation of our study. Moreover, we have not carried on functional studies of the nose after SARME. Our future purpose is to submit a NOSE questionnaire to those patients and evaluate their nasal septum position by nasal fibroendoscopy, to obtain a subjective evaluation of the nasal symptoms reported by the patients, coupled with an objective study of the nasal fossa.

## Conclusion

In surgically assisted maxillary procedures, a mean bony septal deviation ranging from 0.4 to 1.2 mm in any direction can be expected, and this deviation is more pronounced at anterior portion.
